# Mechanical thrombectomy in acute ischaemic stroke patients with pre-interventional intracranial haemorrhage following intravenous thrombolysis

**DOI:** 10.1177/19714009211009112

**Published:** 2021-04-12

**Authors:** Hanna Styczen, Matthias Gawlitza, Nuran Abdullayev, Alex Brehm, Carmen Serna-Candel, Sebastian Fischer, Johannes Gerber, Christoph Kabbasch, Marios-Nikos Psychogios, Michael Forsting, Hans Henkes, Volker Maus

**Affiliations:** 1 Institute for Diagnostic and Interventional Radiology and Neuroradiology, University Hospital Essen, Germany; 2 Institute of Neuroradiology, University Hospital Carl Gustav Carus, Germany; 3 Department of Diagnostic and Interventional Radiology, University Hospital Cologne, Germany; 4 Department of Neuroradiology, University Hospital Basel, Switzerland; 5 Neuroradiological Clinic, Klinikum Stuttgart, Germany; 6 Department of Radiology, Neuroradiology and Nuclear Medicine, Ruhr-University Bochum, Germany

**Keywords:** Acute ischaemic stroke, intracranial haemorrhage, intravenous thrombolysis, large vessel occlusion, mechanical thrombectomy

## Abstract

**Background:**

Data on outcome of endovascular treatment in patients with acute ischaemic stroke due to large vessel occlusion suffering from intravenous thrombolysis-associated intracranial haemorrhage prior to mechanical thrombectomy remain scarce. Addressing this subject, we report our multicentre experience.

**Methods:**

A retrospective analysis of consecutive acute ischaemic stroke patients treated with mechanical thrombectomy due to large vessel occlusion despite the pre-interventional occurrence of intravenous thrombolysis-associated intracranial haemorrhage was performed at five tertiary care centres between January 2010–September 2020. Baseline demographics, aetiology of stroke and intracranial haemorrhage, angiographic outcome assessed by the Thrombolysis in Cerebral Infarction score and clinical outcome evaluated by the modified Rankin Scale at 90 days were recorded.

**Results:**

In total, six patients were included in the study. Five individuals demonstrated cerebral intraparenchymal haemorrhage on pre-interventional imaging; in one patient additional subdural haematoma was observed and one patient suffered from isolated subarachnoid haemorrhage. All patients except one were treated by the ‘drip-and-ship’ paradigm. Successful reperfusion was achieved in 4/6 (67%) individuals. In 5/6 (83%) patients, the pre-interventional intracranial haemorrhage had aggravated in post-interventional computed tomography with space-occupying effect. Overall, five patients had died during the hospital stay. The clinical outcome of the survivor was modified Rankin Scale=4 at 90 days follow-up.

**Conclusion:**

Mechanical thrombectomy in patients with intravenous thrombolysis-associated intracranial haemorrhage is technically feasible. The clinical outcome of this subgroup of stroke patients, however, appears to be devastating with high mortality and only carefully selected patients might benefit from endovascular treatment.

## Background

Mechanical thrombectomy (MT) in combination with intravenous thrombolysis (IVT) is the standard treatment for patients suffering from acute ischaemic stroke (AIS) due to intracranial large vessel occlusion (LVO) in the anterior circulation.^1,2^ The particular benefit of IVT in these patients is unknown. As a result, various randomised controlled studies are currently being conducted to determine if MT without IVT is equally effective (SWIFT-DIRECT, NCT03192332; MR CLEAN NO IV, ISRCTN80619088; DIRECT MT, NCT03469206). Recently, the randomised DIRECT-MT trial in China indicated that MT was noninferior to the combined treatment.^3^ However, the ongoing SKIP trial in Japan could not establish that skipping IVT was noninferior to the combined approach but was at least associated with a lower risk of intracranial haemorrhage (ICH).^4^

Symptomatic intracerebral haemorrhage is the main intracranial complication of IVT with rates reported up to 8.8% according to the European Cooperative Acute Stroke Study II (ECASS II) trial and associated with high mortality rates.^5,6^ Nevertheless, in primary stroke centres (PSCs), the early initiation of bridging therapy remains the only treatment for patients presenting with AIS before admitting these patients to a MT-capable comprehensive stroke centre (CSC). With increasing numbers of patients treated under the ‘drip and ship’ paradigm, the occurrence of an IVT-associated ICH prior to the endovascular procedure is becoming more likely.^7^ Studies analysing the benefit of MT in this subgroup are limited as pre-interventional ICH remains an exclusion criterion for endovascular therapy in clinical trials.^8^ Therefore, we aimed to report our multicentre experience with MT in patients with AIS due to LVO suffering from IVT-associated ICH.

## Methods

We conducted a retrospective study of AIS patients undergoing MT at five tertiary care centres in Germany between January 2010–September 2020.

All patients included in the study were treated with MT due to LVO despite the occurrence of an ICH after initiation of IVT. Inclusion criteria were missing evidence of ICH on baseline imaging, application of IVT and execution of additional imaging prior to the intervention (e.g. due to deterioration of clinical symptoms) using multi-detector or flat-detector computed tomography (CT) with detection of a newly delimited ICH. Extent or location of ICH (parenchymal, subdural or subarachnoid) were not exclusion criteria. Space-occupying effect of a parenchymal haemorrhage was defined as any mass effect on adjacent brain structures such as deep grey matter and gyri with narrowing of sulci, midline shift or brain herniation. Early ischaemic damage was evaluated using Alberta Stroke Program Early CT Score (ASPECTS) on first imaging for the anterior and posterior circulation. In post-interventional ASPECTS areas of intraparenchymal haemorrhage in the affected territory were included in the assessment. Large vessel occlusion was defined as any occlusion in cerebral arteries including distal internal carotid artery (ICA), middle cerebral artery (MCA; M1 and M2 segments), distal vertebral artery, basilar artery (BA) and posterior cerebral artery (P1 segment). Patient demographics, medical history, technical features, angiographic and clinical outcome were noted. The aetiology of the occlusion was based on the Trial of ORG 10172 in Acute Stroke Treatment (TOAST) classification. In addition, the underlying aetiology for ICH (e.g. neoplasm, aneurysm, cavernoma) and localization of the ICH (in/outside the LVO affected territory) was reviewed. Any progression in size with consecutive increase of perifocal oedema was defined as aggravation of the ICH.

All patients received IVT and were treated according to the widely accepted selection criteria with a weight-based infusion of alteplase at 0.9 mg/kg over 60 min with a maximum dose of 90 mg. Ten per cent of the total treatment dose was given as a bolus over 1 min. There were no limitations on procedural characteristics including the use of different thrombectomy techniques, which were left to the attending neuroradiologist’s discretion. Endovascular treatment was performed with approved MT devices using stent-retrievers, large-bore aspiration catheters or a combination of both.

Complete reperfusion was defined as the Thrombolysis In Cerebral Infarction (TICI) scale score of three. Successful reperfusion was defined as TICI≥2b. Clinical efficacy outcome was the rate of functional independence measured by the modified Rankin Scale (mRS) and defined as 0–2 at discharge and 90 days. National Institutes of Health Stroke Scale (NIHSS) and mRS grades were assessed by a consultant neurologist. Baseline NIHSS was collected at patients’ admission at the CSC.

According to the guidelines of the respective local ethics committees, ethical approval was given when necessary for this anonymous retrospective study, which was conducted in accordance with the Declaration of Helsinki. A patient’s consent for treatment was obtained according to the individual institutional guidelines. Due to the retrospective nature of the study, additional informed consent was deemed unnecessary.

## Results

In total, six patients from five tertiary stroke centres were treated with MT due to LVO suffering from IVT-associated ICH. Procedural characteristics per case are shown in Table 1. Out of six patients, five patients received IVT at a PSC and were subsequently transferred to a CSC for endovascular treatment (‘drip-and-ship’ paradigm). One patient was directly transferred to a CSC (‘mothership’ paradigm’). Alteplase was administered as the full dose in patients treated by the ‘drip-and-ship’ paradigm. Median age was 80 years (interquartile range (IQR) 76–85 years) and 4/6 (67%) patients were female.

**Table 1.** Detailed demographic, procedural and outcome parameters.

Case	Sex/age	Baseline medication	Drip and ship	Occlusion site	TOAST	Underlying aetiology for ICH	Baseline ASPECTS	NIHSS admission	Localization of ICH	ICH within the LVO affected territory	Onset to groin (min)	Onset to IVT (min)	Onset to final reperfusion (min)	Final TICI	Number of manoeuvres	mRS 90 days
1	M/78	Aspirin	Yes	M1	Undetermined	Unknown	8	22	Intraparenchymal	No	149	45	193	2a	2	6
2	F/85	Aspirin	Yes	BA	Cardioembolic	Unknown	8	22	Intraparenchymal and subdural	Yes	NA	NA	NA	2b	3	6
3	F/86	No	Yes	Distal ICA	Cardioembolic	Vital cancer	10	30	Intraparenchymal	Yes	230	80	335	2b	3	6
4	M/70	Aspirin	Yes	M1	Undetermined	Unknown	8	20	Intraparenchymal	Yes	240	150	306	2b	1	4
5	F/79	No	No	BA	Cardioembolic	Unknown	8	12	Intraparenchymal	Yes	320	60	660	2a	8	6
6	F/80	Aspirin	Yes	BA	Large artery Sclerosis	Unknown	10	36	Subarachnoid	Yes	288	179	358	2b	1	6

ASPECTS: Alberta Stroke Program Early CT score; BA: basilar artery; F: female; ICA: internal carotid artery; ICH: intracranial haemorrhage; IVT: intravenous thrombolysis; LVO: large vessel occlusion; M: male; min: minutes; mRS: modified Rankin Score; NIHSS: National Institutes of Health Stroke Scale; SAH: subarachnoid haemorrhage; TICI: Thrombolysis In Cerebral Infarction.

**Figure 1.**
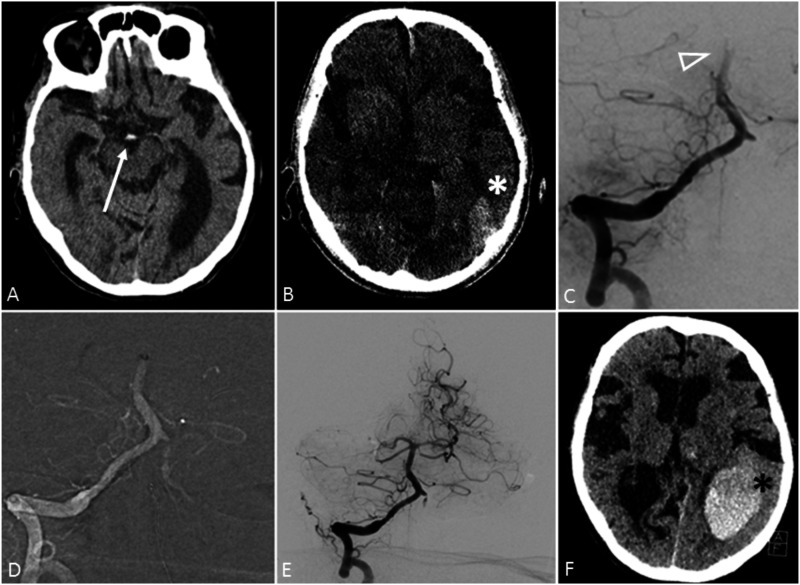
(a) Baseline imaging from an octogenarian woman with basilar artery occlusion and dens artery sign (white arrow) on non-contrast computed tomography. Intravenous thrombolysis was given, and the patient was transferred to a comprehensive stroke center. (b) At the beginning of the endovascular procedure a flat detector computed tomography was done due to deterioration of symptoms (National Institute of Health Stroke Scale=22) with evidence of an intraparenchymal haemorrhage in the left occipital lobe (white asterisk). At this timepoint, alteplase was already administered completely. (c)–(e) Mechanical thrombectomy was done with successful recanalization of the basilar artery. The right posterior cerebral artery was chronically occluded. (f) Computed tomography 24 h later showed an aggravation of the intracranial haemorrhage (black asterisk). The patient died due to respiratory failure during the hospital stay.

Large vessel occlusion of the BA was detected in 3/6 (50%) patients, two patients suffered from MCA M1 and one patient from distal ICA LVO, respectively. Five out of six (83%) individuals demonstrated cerebral intraparenchymal haemorrhage on pre-interventional CT. Of those, 3/5 (60%) patients suffered from space-occupying haematoma. In one patient, additional subdural haematoma was observed, and one patient suffered from isolated subarachnoid haemorrhage (SAH). Five out of six (83%) ICH events were localised in the LVO affected territory (Figure 1).

Cardioembolic cause was the most common aetiology for the LVO and found in 3/6 (50%) patients, followed by large artery atherosclerosis (1/6; 17%). Stroke aetiology remained unknown in two patients. The underlying aetiology for the ICH remained undetermined in all patients except for one, for whom further work-up revealed vital cancer, which might have increased the bleeding propensity. Four out of six patients had previous medication with antiplatelet agents.

Median baseline NIHSS at CSC admission was 22 and median baseline ASPECTS on first imaging was eight. The initial NIHSS at the primary stroke centre was not documented in 5/6 patients. The median interval between (a) onset and IVT was 80 min (IQR 53–165 min), (b) IVT and groin puncture was 107 min (IQR 94–193 minutes) and (c) onset and groin puncture was 240 min (IQR 190–340 min), respectively. The rate of pre-treatment functional independence (mRS≤2) was 50% (3/6).

### Procedural and functional outcome

The median time interval from groin puncture to final reperfusion was 70 min (IQR 61–146 min) and the median number of thrombectomy manoeuvres was three (range 1–8). Successful reperfusion was achieved in 4/6 (67%) patients. None of the six patients were reperfused completely. The median ASPECTS in post-interventional CT was six. In the majority of cases (5/6, 83%), the IVT-associated ICH had aggravated in post-interventional imaging with space-occupying oedema.

**Figure 2.**
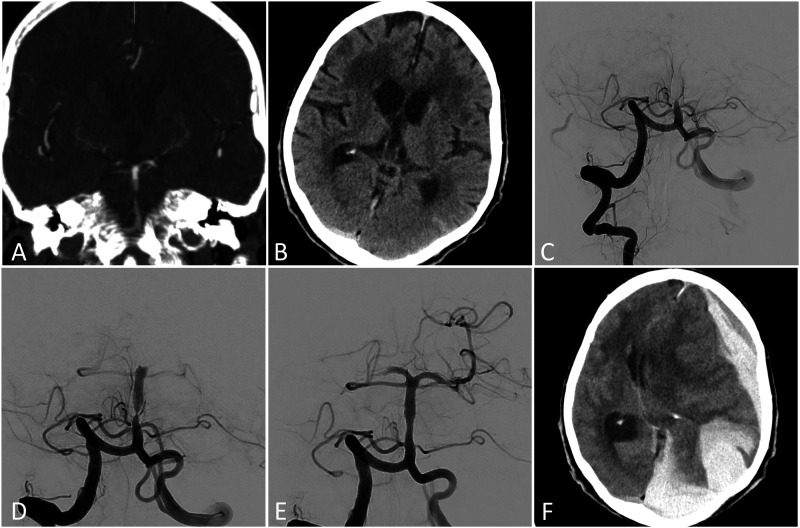
An 80-year-old female patient with acute basilar artery occlusion, treated with intravenous thrombolysis at the referring centre (not shown). Upon arrival computed tomography showed a persistent proximal basilar artery thrombosis with reperfusion of the basilar apex (a). Native computed tomography demonstrated a slight subarachnoid haemorrhage in the left temporal region (b). The first angiogram showed persisting occlusion of the proximal third of the basilar artery (c). After one aspiration the vessel was recanalised, but revealed a proximal high-grade stenosis (d), which re-occluded 10 min later. The implantation of a self-expanding stent with following percutaneous transluminal angioplasty led to good reconstitution of the vessel (e). Recanalization of the occluded right P2 segment was not attempted. As the patient was already pretreated with a daily dose of 100 mg of aspirin, an intravenous infusion of tirofiban was initiated with the intention to load the patient with clopidogrel in an overlapping fashion. Four hours later the patient demonstrated wide and fixed pupils bilaterally. An emergency computed tomography scan showed a massive haemorrhage with intraparenchymal, subarachnoid and subdural components. Surgical evacuation was not attempted, and the patient died 2 days later.

Procedure-related, minor SAH had occurred in 1/6 (17%) patients. In one patient, intracranial stenting was performed (Case 6). The patient presented with an occlusion of the proximal BA at a PSC (Figure 2) and after IVT was administered, the patient was transferred to the CSC. As the patients’ status had become impaired, CT imaging was done at the CSC with evidence of slight SAH. The patient was transferred in the angiography suite and the occlusion was recanalised successfully with one aspiration attempt. However, a high-grade stenosis was confirmed in the proximal BA segment, which re-occluded instantly. The operator decided to implant a self-expanding stent with subsequent balloon angioplasty, resulting in good reconstitution of the vessel. As the patient was already pretreated with a daily dose of 100 mg of aspirin, an intravenous infusion of tirofiban was initiated. Four hours later the patient demonstrated wide and fixed pupils bilaterally. An emergency CT scan showed a massive haemorrhage with intraparenchymal, subarachnoid and subdural components. Surgical evacuation was not attempted, and the patient died 2 days later.

Overall, five patients had died during hospital stay. The survivor initially presented with a non-space occupying ICH and achieved a complete resorption of the haemorrhage at discharge; at 90 days, the clinical outcome was moderate an mRS=4 (pre-treatment mRS=2, Case 4).

## Discussion

We provide the first report of a series of AIS patients with LVO suffering from IVT-associated ICH prior to MT. Our study revealed several findings: (a) the procedure is technically feasible with an adequate rate of successful reperfusion, (b) the mortality rate of these subgroup of patients is high (83%), and (c) the underlying cause for the ICH remains undetermined in most cases.

As the presence of an intracranial haemorrhage currently represents an exclusion criterion for MT, the decision-making was done individually. In three of the affected individuals, the haemorrhage was not space-occupying (Cases 2, 4 and 6). One patient showed haemorrhage outside the affected territory (Case 1). One patient suffered from a basilar artery occlusion and clinical outcome is known to be devastating if occlusion will not be recanalised (Case 5). In another patient (Case 3), it was the decision of the operator and the neurologist as the patient was in her 80s, but functionally independent prior to the stroke.

So far, there is one case report about a successful MT in a 75-year-old patient with MCA occlusion and pre-interventional IVT-associated ICH.^8^ The underlying cause for the ICH was probable cerebral amyloid angiopathy and the patient was released with an excellent neurological outcome (mRS=0). In comparison with our study, the patient had lower NIHSS at admission (NIHSS=7) and was reperfused completely. In addition, the IVT-associated ICH appeared not to be space-occupying.

The rate of successful reperfusion in our study was lower compared to the current literature^5^ and might be due to several factors. First, the median age of the included patients was 80 years and all patients had arterial hypertension with increased likelihood of difficult vascular access resulting in longer procedure times, lower recanalization rates and poorer outcome.^9^ Second, half of the occluded vessels were localised in the posterior circulation. A recent study reported that futile recanalization occurred more frequently in BA occlusions, and predictors of futile recanalization included age, stroke severity, manoeuvre count and intracranial stenting.^10^

In our study, the mortality rate was high with 83% compared to patients with LVO receiving IVT in the anterior circulation with rates up to 15% in the interventional arm (MT and IVT) of the HERMES meta-analysis.^5^ It remains unclear whether the devastating clinical outcome in our study was caused by the LVO or the ICH itself. Since the ICHs were space-occupying in the majority of cases, it might have been attributed most likely to the neurological aggravation. However, it has to be mentioned that two of the patients were not reperfused successfully (TICI=2a each), which is also accompanied with poor clinical outcome.^11^ In contrast to the deceased, the survivor in our cohort suffered from an M1 occlusion and a small parenchymal haemorrhage, which had resorbed completely at discharge. Although two other patients also showed non-space-occupying ICHs on pre-interventional CT, the clinical outcome was poor due to underlying BA occlusion and an aggravation of the ICHs during the hospital stay. Although the presented number of patients is low, it is conceivable that the extent of baseline ICH and expansion after MT might influence patients’ outcome.

In this context, blood pressure (BP) control might be one important factor affecting the outcome. On the one hand, moderately elevated BP is associated with good collateral circulation in AIS patients.^12^ On the other hand, high BP levels around reperfusion therapy carry an increased risk of ICH.^13^ In the status of vessel occlusion, low BP levels may lead to hypoperfusion of ischaemic tissue resulting in greater infarction.^14^ In the unlikely event of both, ICH and simultaneous cerebral LVO, the optimal target BP remains indeterminable, since both variations provoke poor neurological outcome.

In a meta-analysis risk factors for ICH in AIS patients treated with IVT were identified including higher age, higher stroke severity and higher glucose level.^15^ The study showed that there was approximately a doubling of the odds of ICH with the presence of a visible acute cerebral ischaemic lesion on pretreatment brain imaging.^15^ Some of these baseline factors are also reflected in our cohort regarding high median age, high median baseline NIHSS, a median ASPECTS=8 on baseline imaging, previous antiplatelet agents and comorbidities. From this point of view, it might be reasonable in this subgroup of patients, and more particularly in patients treated by the ‘drip and ship’ paradigm, to enforce a flat detector CT pre-interventionally in case of deterioration of the patients’ status.

The underlying cause for the IVT-related ICH was undetermined in most cases. This might be due to the fact, that in most patients advanced imaging including post-interventional magnet resonance imaging has not been executed.

In patients with LVO based on an intracranial atherosclerotic disease the treatment of the underlying stenosis is challenging. In our study, one individual suffered from a slight pre-interventional SAH and an acute high-grade stenosis of the BA. At this point, the risk of progressive bleeding due to the necessity of potent antiplatelet medication must be weighed against re-occlusion of the vessel. The patient in our study was treated with intracranial stenting and peri-interventional administration of tirofiban, which led to a massive intracranial haemorrhage. In these specific cases, alternatively a percutaneous transluminal balloon angioplasty might be a reasonable treatment option with waiving of glycoprotein IIB/IIIA inhibitors administration.

The main limitation of our study is the retrospective nature including inherent selection bias and the usage of different thrombectomy equipment and techniques. The lack of a control group is a further limitation. As highlighted previously, the presented number of patients is too low to draw definitive conclusions, especially as half of the patients suffered from BA occlusion, which by itself is accompanied with a poorer outcome compared to anterior circulation strokes and might serve as a possible confounder. However, this multicentre study includes the largest number of endovascularly treated LVOs following IVT-associated ICHs in the literature so far.

## Conclusion

Mechanical thrombectomy in patients with IVT-associated ICH is technically feasible. The clinical outcome of these patients appears to be devastating with high mortality and only carefully selected patients might benefit from endovascular treatment. However, until we have data from larger trials, it will always be an individual decision but, finally, it should be the goal to not leave individuals behind that might benefit from endovascular treatment in this particular situation.
